# Monitoring transient nanoparticle interactions with liposome-confined plasmonic transducers

**DOI:** 10.1038/micronano.2016.86

**Published:** 2017-04-10

**Authors:** Tianhong Chen, Xiao Wang, Mohammad Hossein Alizadeh, Björn M. Reinhard

**Affiliations:** 1 Department of Chemistry and The Photonics Center, Boston University, 590 Commonwealth Avenue, Boston, MA 02215, USA

**Keywords:** gold nanoparticles, molecular rulers, plasmon, plasmon coupling, plasmon hybridization, plasmon rulers, self-assembly

## Abstract

The encapsulation of individual pairs of plasmonic nanoparticles (NPs) in liposomes is introduced as a new strategy for utilizing plasmon coupling to monitor interactions between co-confined NPs in a nanoconfinement that ensures high local NP concentrations. We apply the approach to monitor transient binding contacts between noncovalently tethered 55 nm diameter gold NPs, which were functionalized with cytosine (C)-rich DNAs, in acidic and mildly basic buffer conditions. At pH=8, a rich spectral dynamics indicates DNA-mediated transient binding and unbinding of co-confined NPs due to weak attractive interparticle interactions. A decrease in pH from 8 to 4 is observed to favor the associated state for some co-confined NPs, presumably due to a stabilization of the bound dimer configuration through noncanonical C-C^+^ bonds between the DNA-functionalized NPs. Plasmonic nanoemitters whose spectral response switches in response to chemical cues (in this work pH) represent optical transducers with a rich application space in chemical sensing, cell analysis and nanophotonics.

## Introduction

Light incident on gold and silver nanoparticles (NPs) can excite coherent oscillations of conduction band electrons in the visible range of the electromagnetic spectrum. These surface charge density oscillations are referred to as localized surface plasmon resonances and form the basis for several imaging and sensing applications^1–3^. In a dimer of NPs, the electric (*E*−) fields associated with the surface electron density oscillations induce an effective coupling of the plasmons. Because the coupling strength depends on the interparticle separation, the NP dimers show a distance-dependent plasmon resonance^4–14^. The plasmon resonance wavelength of plasmonic ‘molecules’ can be conveniently monitored in the far field^15,16^. Consequently, discrete pairs of biopolymer-tethered NPs represent nanoscale distance transducers that are commonly referred to as plasmon rulers (PRs)^6,9,17^. Because of the exceptional photostability and large resonant scattering cross-sections of NPs, PRs can overcome limitations associated with blinking, bleaching, and low signal to noise that plague alternative organic-dye-based ruler technologies. However, PR applications are not limited to distance sensing; it was recently demonstrated that the continuous thermal interparticle fluctuations in PRs contain information about the interparticle potential and tether stiffness^18^. New applications of PRs, for instance, as optical detectors of coherent charge transport across molecules or reversible optical switches, continue to emerge^19,20^. Therefore, PRs and related plasmonic transducers are actively pursued in a wide range of technological and sensing applications^18,20–32^. In particular, the ability to perform single PR measurements in a massively parallel manner is of significant interest to develop single-molecule assays that can record statistically meaningful single-molecule data in a time-efficient manner^21,33^. In most cases, the tether in PRs is a nucleic acid such as DNA or RNA. These PRs have been applied to monitor distance changes in response to buffer changes^17^, enzyme binding^33^, tether cleavage^26,27^ or to monitor the dynamics of membrane compartments in living cells^34^. The introduction of nucleic acid aptamers as the tether molecule has further broadened the application space of PRs in ultrasensitive biosensing and metabolomics^24^.

Many sensor applications of PRs require their surface immobilization to eliminate diffusion and facilitate long observation times. However, interactions between NPs and the surface can perturb the structure, functionality and dynamics of the PRs. Furthermore, weak interactions between functionalized NPs may lead to only transient contacts between the NPs. The dependence of transient contacts on details of the NP surface and ambient medium are of fundamental interest but impossible to study with conventional PR techniques, as the latter require a stable tether between the individual NPs.

In fluorescence microscopy, the great value of dye-based molecular rulers (that is, fluorescence resonance energy transfer)^35^ as a unique tool to overcome ensemble averaging and identify transient intermediates is well recognized^36^. In this context, liposome confinement strategies for fluorescently tagged single molecules have been developed to (i) enable the measurement of weak and transient contacts and (ii) minimize the detrimental surface effects when surface immobilization is required to achieve observation times that are much longer than typical diffusion times^37,38^. Inspired by this approach, we implement in this manuscript a liposome confinement approach for metal NP dimers and larger clusters that does not require a stable tether between the NPs. This approach retains the NPs suspended in the liposome interior, where they are protected from the perturbations of the supporting surface. By co-confining multiple NPs to the interior of one liposome, NPs can be maintained in close vicinity for extended periods of time without a molecular tether between the NPs. The spatial co-confinement, together with the unlimited lifetime of the NP probes, provides unique opportunities for monitoring weak and/or transient NP interactions without limitations in observation time through distance-dependent plasmon coupling. In the following sections, we will apply this approach to test the pH dependence of cytosine-mediated interactions between DNA-functionalized gold NPs (DNA-NPs).

## Materials and methods

### Preparation of DNA-NPs

Citrate-stabilized gold NPs (55.4±1.4 nm *Z*-average diameter) were obtained via a Turkevich synthesis^39^. First, the gold colloid with a concentration of ~9×10^10^ particles per mL was stabilized by adding bis(*p*-sulfonatophenyl)-phenylphosphine (BSPP) dipotassium salt at a concentration of 1 mg mL^−1^ and incubated in a 45 °C water bath overnight. Then, the particles were washed once by centrifugation (1500 *g*, 10 min) and redispersed in T10 (10 mM NaCl/10 mM Tris, pH 8.0) to a final concentration of ~3×10^12^ particles per mL. To prepare DNA-NPs, the particles were incubated with thiolated single-stranded C-rich oligonucleotides (DNA) with the sequence 5′-HS-GATCCTATAAAACCCCAACCCC-3′ (Integrated DNA Technologies, Coralville, IA, USA). Five μL of 1 μM DNA was added into each centrifuge tube with 30 μL of concentrated NP solution and incubated overnight at room temperature. Subsequently, 3 μL of a 2% (w/w) thiol-alkyl-PEG-COOH (polyethylene glycol-functionalized NP (PEG-NPs); HS-C_11_H_22_-EG_6_-COOH, EG=(OCH_2_CH_2_); NANOCS Inc., New York, NY, USA) aqueous solution was added and incubated overnight to further stabilize the gold NPs. Control NPs coated only with PEG-NPs were obtained by directly incubating NPs with PEGs overnight after BSPP functionalization and buffer exchange into T10. All DNA-NPs were washed three times by centrifugation (1500 *g*, 10 min) and redispersed in distilled deionized water. Then, the NPs were pelleted by centrifugation and redispersed in P50 (50 mM NaCl/10 mM citrate-phosphate, pH 8.0) to a final concentration of ~3×10^12^ particles per mL. The *Z*-average diameters were determined as 60.48±0.37 and 61.28±0.15 nm for PEG-NPs and DNA-NPs, respectively (dynamic light scattering (DLS)). All colloid dispersions were stored at 4 °C and used within two days of preparation.

### Preparation of NP-liposomes

NP-liposomes were prepared using a reverse-phase evaporation method^40^ to ensure maximum encapsulation of NPs into liposomes. One hundred μL of DNA-NPs (3 nM) were combined with 200 μL of a 12.5 mg mL^−1^ lipid solution in chloroform. The lipid mixture contained 98% (w/w) 1-palmitoyl-2-oleoyl-*sn*-glycero-3-phosphocholine (POPC) and 2% (w/w) 1,2-dioleoyl-*sn*-glycero-3-phosphoethanolamine-*N*-(cap biotinyl) sodium salt (Avanti Polar Lipids Inc., Alabaster, AL, USA). For the colocalization experiments, the lipid mix also contained 2% (w/w) 1,1′-dioctadecyl-3,3,3′,3′-tetramethylindocarbocyanine perchlorate (DiI; Invitrogen, Carlsbad, CA, USA), and the POPC content was reduced to 96%. The two-phase mixture was sonicated with a probe sonicator for 30 s. Then, chloroform was removed in vacuum on a rotary evaporator. The resulting viscous solution was diluted with P50 buffer and extruded through a 200-nm pore size polystyrene membrane with a mini extruder set (Avanti Polar Lipids Inc.). After the extrusion, the liposome solution was centrifuged at 100 *g* for 30 min to collect the formed NP-containing liposomes.

### Preparation and loading of flow chambers

A microcapillary flow chamber with the dimensions of 0.010×2.00×100 mm (inner diameter×width×length; Vitrocom, Mountain Lakes, NJ, USA) was sequentially incubated with 1 mg mL^−1^ biotinylated bovine serum albumin, 1 mg mL^−1^ Neutravidin and blocking buffer (Superblock; Pierce, Rockford, IL, USA) for 20 min. The superblock was added to minimize any nonspecific binding of liposomes or any remaining free NP monomers. The NP-liposome solution from centrifugation after extrusion was diluted ~20 times with P50 buffer and flushed through the flow chamber. Subsequently, the chamber was washed with clean P50 buffer. Biotin-labeled liposomes were successfully immobilized on the surface by binding to Neutravidin, whereas the free NPs showed only negligible binding.

### Darkfield microscopy

Darkfield experiments were performed with an Olympus IX71 (Olympus, Center Valley, PA, USA) inverted microscope. The samples were illuminated through an oil darkfield condenser (NA 1.2–1.4) with a 100 W Tungsten lamp. Scattering signals from the NP-liposomes in the sample plane were collected with a ×60 oil objective (Olympus RMS60X-PFOD) with NA 0.65. The signal was further magnified by a ×1.6 lens and subsequently split into two beam paths (530 and 585 nm channels) using a 560-nm dichroic mirror and two bandpass filters. The signal of each channel was collected on two separate electron-multiplying charge-coupled devices (Andor IxonEM^+^, Andor, South Windsor, CT, USA) with detection areas of 40×40 μm^2^ and 128×128 pixels resolution. The two cameras were triggered to simultaneously start with an external pulse generator. The exemplary movie in the Supplementary Information was recorded with a digital camera (Nikon 5200, Nikon, Melville, NY, USA) through an eyepiece adaptor.

### Data processing and analysis

Darkfield movies were recorded from 10 different locations in one chamber. The movies were 20 s in length and typically contained tens of NP-liposomes in each field of view. The raw intensity data of each particle in each frame of each channel was fitted with a three-dimensional Gaussian peak, whose integrated volume was used as the particle intensity by a home-written Matlab code^41^.

### Electromagnetic simulations

The Finite Element Method solver COMSOL (COMSOL Inc., Burlington, MA, USA) was used to calculate the radiation pattern of the dimer under each specific polarization of the incident light with an angle of incidence of 60°. The finite difference time domain method was implemented to calculate the optical cross-sections. A fine mesh with a minimum mesh size of 2 nm was used.

## Results and discussion

**Figure 1:**
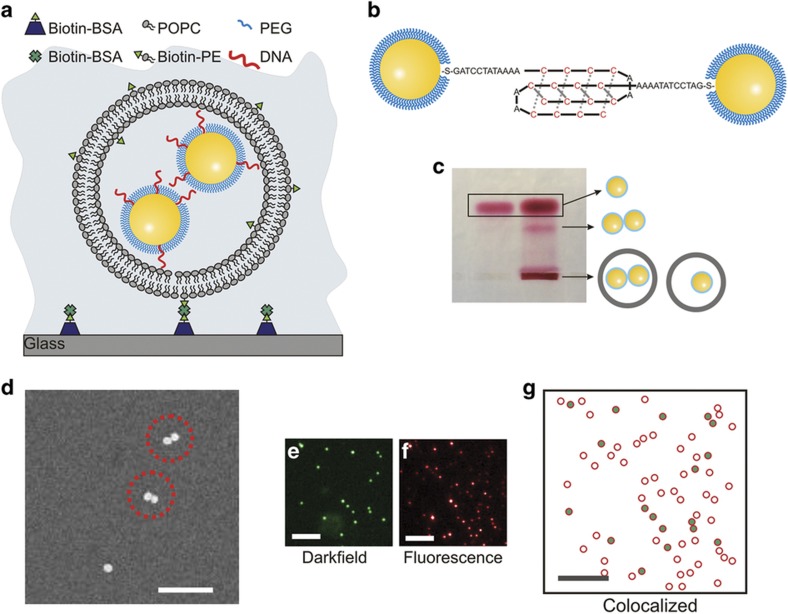
Characterization of NP-liposomes. (**a**) Schematic drawing of liposome-encapsulated NPs (NP-liposome). (**b**) Model of DNA-NPs functionalized with DNA strands that are integrated into a self-assembled monolayer of short PEGs. The cytosine-rich single-stranded DNA strands facilitate the formation of bimolecular i-motif-like binding contacts between DNA-NPs under acidic buffer conditions. (**c**) Right lane: Electrophoresis of NP-liposome in 1% agarose gel. Left lane: DNA-NP controls that are not encapsulated in a liposome. Assignments of the bands are included as schematic drawings. (**d**) SEM pictures of NP-liposomes; scale bar, 350 nm. (**e**) Darkfield scattering image, (**f**) fluorescence image, and (**g**) magnified overlap image of a typical NP-liposome preparation after surface immobilization: the green dots mark the positions of the detected NPs; the red circles show the positions of the liposomes. The scale bars are 10 μm in (**e**–**g**). NP, nanoparticle; PEG, polyethylene glycol; SEM, scanning electron microscopy.

The proposed liposome encapsulation strategy is schematically illustrated in Figure 1a. The few-nanometer-thin lipid bilayer that defines the liposome nanocontainer is assembled predominantly from zwitterionic POPC. Consequently, it has a low effective surface charge under slightly basic pH (*ξ*=−1.11±0.82 mV at pH=8.0) and shows much weaker nonspecific interactions with the NPs than is the case for a conventional glass support. In fact, we did not observe any nonspecific binding of DNA or PEG-NPs to these membranes in a previous study^42^. The superb brightness of liposome-confined plasmonic particles that are immobilized on a glass support provides excellent signal to noise at high acquisition rates^42^. Furthermore, because the NP signal is based on light scattering, the assay is compatible with long observation times and facilitates the systematic investigation of the same pair of NPs co-confined in one liposome under different environmental conditions. The latter is extremely useful for monitoring complex processes free of ensemble averaging over potentially heterogeneous size and shape distributions.

To maximize the NP encapsulation efficiency, we used a reverse-phase evaporation approach to prepare NP-containing liposomes^40^. The 200 nm diameter liposomes generated in this manner had a nominal total volume of 4.2×10^−18 ^L, which compares to ~1.1×10^−19^ L for an NP with a hydrodynamic diameter of 60 nm. The co-confinement of two or more NPs in one liposome generates locally very high NP concentrations (for 2 NPs confined to a 200-nm diameter liposome, the effective concentration is ~5×10^14^ NP per mL) and ensures high interaction probabilities. The co-confinement of the NPs into a small volume makes the system, therefore, suitable for investigating interparticle interactions in the absence of a connecting tether. Furthermore, the liposome-confined NPs can also be applied for characterizing changes of the interparticle potential as a function of environmental conditions, such as ligand binding to the NPs or pH-induced changes in the NP surface chemistry. In particular, we investigated the pH-dependent interparticle interactions between C-rich DNA-NPs that can potentially form bimolecular i-motif^43^-like binding contacts (Figure 1b).

The successful encapsulation of DNA-NPs in the liposomes was verified by gel electrophoresis (Figure 1c). In addition to excess gold NP monomers and dimers, which run in the gel because of their negative charge and give rise to the marked bands, the gel shows significant material remaining in the loading wells. Based on the vividly red color, we can exclude that these NPs are large agglomerates. Instead, the immobile fraction represents successfully encapsulated NPs that do not enter the gel because of the low surface charge of the encapsulating liposomes (*ξ*=−1.11±0.82 mV). Coincidentally, the gel electrophoresis approach is also a convenient method for separating liposome-encapsulated NPs from excess free NPs. Since the latter readily enter the gel after a field is applied, only the immobile liposome-encapsulated NPs remain in the loading wells, where they can be easily collected. We analyzed the recovered NP-liposome sample using scanning electron microscopy (SEM) after immobilization on a silicon substrate (Figure 1d). This analysis reveals that under the selected, non-optimized experimental conditions, the liposomes contained predominantly one (70%) or two (23%) NPs.

We independently verified the successful encapsulation of NPs by optically colocalizing the NP scattering signal from darkfield images (Figure 1e) with the membrane fluorescence of liposomes that contained 2% (w/w) DiI (Figure 1f). The optical measurements reveal that all metal NPs are colocalized with liposomes (Figure 1g). We emphasize that the scattering signal of the NP-liposomes is determined by the plasmonic gold NPs. The background from free liposomes is negligible. Given the weak scattering signal from excess liposomes in darkfield microscopy, we refrained from a further separation of excess liposomes from NP-liposomes.

Near-field coupled NPs show changes in the interparticle separation as a spectral far-field shift^15,16,44^. Our experimental strategy was to use this effect as a transducer to monitor the interparticle separations between the co-confined DNA-NPs in NP-liposomes. To demonstrate the viability of this approach, we implemented a pH-dependent configurational switch, which was formed by C-rich single-stranded DNAs^45,46^. This approach enabled us to modulate the interparticle potential and switch between two different binding affinities. We passivated NPs with a self-assembled brush of thiol-alkyl-PEG-COOH (simply referred to as ‘PEG’ in the following), in which we integrated ~30 copies of C-rich single-stranded DNAs. Throughout the manuscript the DNA-functionalized NPs are referred to as DNA-NP and PEG controls as PEG-NP. Cytosine has a p*K*_a_ value of 4.4 and is partially protonated under mildly acidic pH values. The partial protonation reduces the surface charge of the negatively charged DNA and facilitates hemiprotonated C-C^+^ base pairing. Both effects together can favor the formation of bimolecular i-motif-like binding contacts between the NPs (Figure 1b)^43^, which are expected to decrease the average separation between the co-confined NPs and result in a measurable spectral redshift.

**Figure 2:**
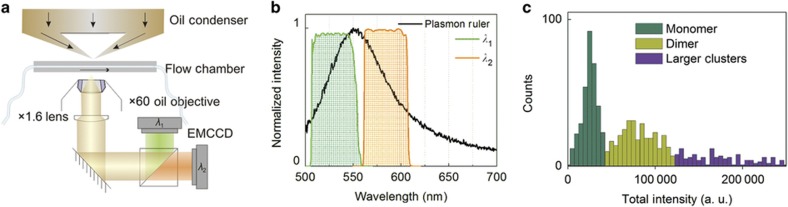
Experimental setup. (**a**) A microfluidic chamber containing surface immobilized NP-liposomes was mounted in a darkfield microscope with ratiometric imaging capability. (**b**) Spectrum of a near-field coupled NP dimer (plasmon ruler) and spectral range of the bandpass filters for the monitored intensity channels *I*(*λ*_1_) and *I*(*λ*_2_). (**c**) Total intensity (*I*(*λ*_1_)+*I*(*λ*_2_)) distribution of NP-liposomes with different numbers of encapsulated NPs. NP, nanoparticle.

Biotin-presenting NP-liposomes were tethered to the interior surface of a Neutravidin-functionalized flow chamber, which was mounted in a darkfield microscope. The flow chamber facilitated a rapid exchange of the buffer using a vacuum flow system and provided easy control of the ambient pH. We used a fast ratiometric imaging approach to monitor the spectral response upon change of the pH for many individual surface-tethered NP-liposomes simultaneously^18,44^. This technique is based on conventional darkfield widefield microscopy, where the entire field of view is illuminated with unpolarized white light at oblique angles, and the signal scattered from any point in the field of view is detected on two separate wavelength channels, *λ*_1_ and *λ*_2_ (Figure 2a). Our experimental setup facilitated the collection of *I*(*λ*_1_) and *I*(*λ*_2_) intensities in an active area of 40 μm by 40 μm with a frame rate of 490 frames per second. The monitored wavelength channels *λ*_1_ and *λ*_2_ were located on the low- and high-energy sides, respectively, of the plasmon resonance wavelength of individual NPs: *λ*_1_=530±21.5 nm and *λ*_2_=585±20 nm (Figure 2b).

Because of the small size of the liposomes and the rapid motion of the NPs within the liposomes, it is not possible to optically discern or localize individual NPs in the confinement. However, spectral shifts due to an enhanced plasmon coupling are detectable as a change in the relative intensities, evaluated as the intensity ratio *R*=(*I*(*λ*_1_)−*I*(*λ*_2_))/(*I*(*λ*_1_)+*I*(*λ*_2_)) of the two monitored wavelength channels. A decrease in *R* indicates a spectral redshift, whereas an increase in *R* indicates a blueshift.

Figure 2c contains the histogram of the total intensity (*I*(*λ*_1_)+*I*(*λ*_2_)) distribution (the intensities were averaged over 20 s long movies) for ~830 NP-liposomes at pH=8. The histogram shows two peaks, which correspond to liposomes with one (first peak) or two (second peak) NPs, and a tail generated by larger clusters. We only include trajectories with average intensities in the NP dimer range in our subsequent analyses.

**Figure 3:**
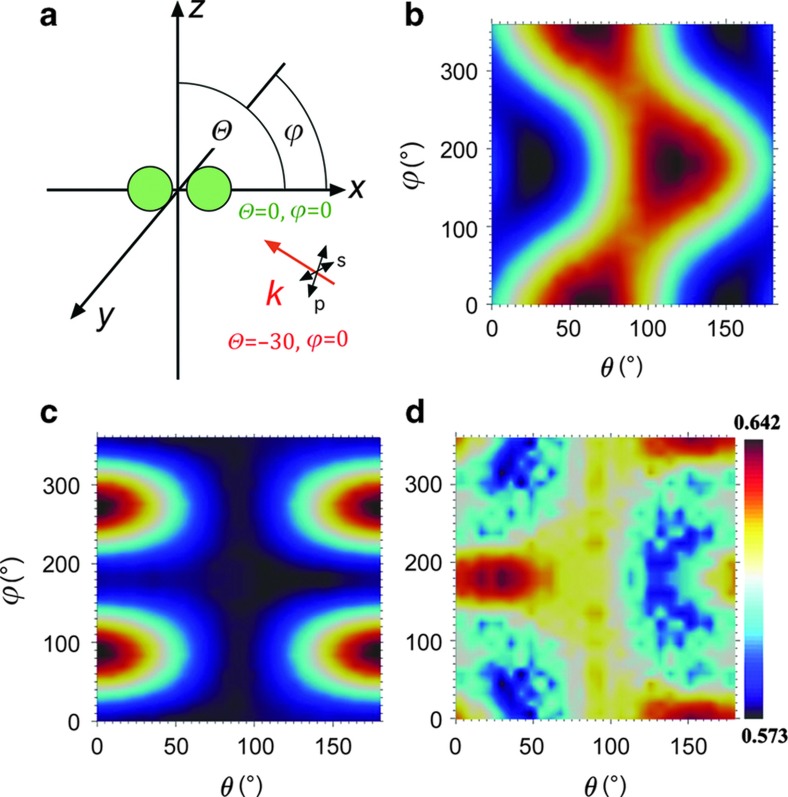
Dependence of the spectral response on the NP dimer orientation. (**a**) Geometric details of the simulations. The dimer comprises a pair of gold monomers, each of which had a diameter of 60 nm and was separated from each other by 10 nm. The long axis of the dimer was aligned with the *x* axis, and the light was incident with an oblique angle of *θ*=−30°; *θ* and *φ* are the polar and azimuthal angles in the text. (**b**) Calculated extinction cross-section at 530 nm for p-polarized light. (**c**) Calculated extinction cross-section at 530 nm for s-polarized light. (**d**) Normalized difference in polarization-averaged cross-section Δ*σ*_norm_ between *λ*=530 and 585 nm as a function of *θ* and *φ*. NP, nanoparticle.

The spectrum of the NP dimers confined to the liposomes can switch between two distinct states with characteristic *R* values, a bound state that gives rise to strong plasmon coupling and an unbound state with little to no effective coupling. In principle, the far-field spectrum of an NP dimer that can rotate in space is, however, not entirely determined by the interparticle separation but also depends on the orientation of the long dimer axis relative to the incident light polarization^47^. To estimate the magnitude of this effect, we evaluated the orientation dependence of the NP dimer signal under oblique illumination as follows. We calculated the extinction cross-sections for a gold dimer with constant interparticle separation (edge-to-edge separation: 10 nm) for different combinations of azimuthal and polar angles (*θ* and *φ* as defined in Figure 3a) at two wavelengths (*λ*=530 and 585 nm) for s- and p-polarized light. The azimuthal angle is measured in the *xy* plane in a counterclockwise manner from the *x* axis. Similarly, the polar angle is measured in the *xz* plane with respect to the *x* axis in a counterclockwise manner. The light was incident at *θ*=−30°. Figures 3b and c show the obtained contour plots for p- and s-polarized light at *λ*=530 nm. In the case of p-polarized light, the spectrum demonstrates two distinctive peaks for (*θ*=60°, *φ*=0°) and (*θ*=120°, *φ*=180°). Because of the rotational symmetry of the dimer in the *xy* plane, the angle designated by (*θ*=60°, *φ*=360°) is identical to (*θ*=60°, *φ*=0°). For the first unique maximum at (*θ*=60°, *φ*=0°), the long axis of the NP dimer is aligned parallel to the *E*-field of the incoming p-polarized light, which ensures an efficient excitation of the longitudinal dimer mode. The peak at (*θ*=120°, *φ*=180°) corresponds to the parallel alignment of the incident *k*-vector with the long dimer axis. In this configuration the excitation of the vertical plasmon mode in the dimer is most efficient. Figure 3c is identical to Figure 3b, but for an s-polarized incident light. Here the peaks occur at (*θ*=0°, *φ*=90°) and (*θ*=0°, *φ*=270°) when the dimer lies in the *xy* plane with its long axis parallel to the electric field of the s-polarized wave.

The effective cross-sections of the dimer for unpolarized light excitation at the investigated wavelengths of *λ*=530 and 580 nm were obtained through incoherent averaging of the calculated cross-section for s- and p-polarizations. The difference between the polarization-averaged cross-sections normalized by their sum generates a dimensionless number Δ*σ*_norm_, which is equivalent to the experimental *R* ratio. Importantly, the simulated Δ*σ*_norm_ values (Figure 3d) remain notably close to 0.5 with a dynamic range <0.070 for all possible *θ*=[0–180°] and *φ*=[0–360°] combinations. It is conceivable that the simulated Δ*σ*_norm_ values differ from the exact experimental *R* values as interparticle separation, size and shape of the NPs as well as the refractive index of the ambient medium (we assumed *n*=1 in our simulations) need to be specified in the simulations. However, the small dynamic range of Δ*σ*_norm_ for different (*θ*, *φ*) configurations found in our simulations confirms unambiguously that the orientation dependence of the spectral dimer response is much weaker than the well-known distance dependence for the chosen NPs^7,9,14^.

**Figure 4:**
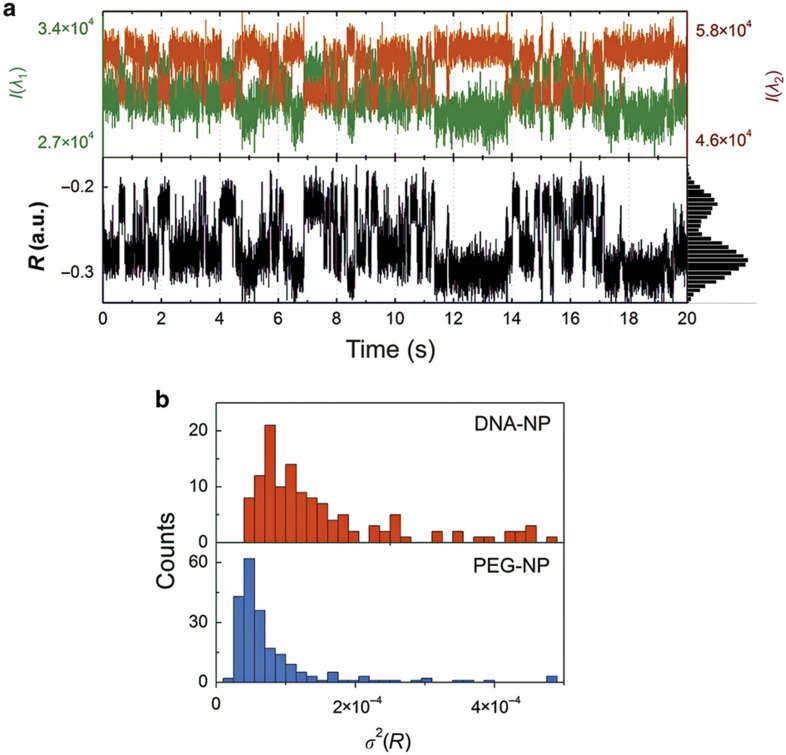
Spectral fluctuation in NP-liposomes that contained two co-confined NPs. (**a**) Scattering trajectories for a NP-liposome with DNA-NPs at pH=8. The top panel shows the scattering intensities recorded on the *I*(*λ*_1_) and *I*(*λ*_2_) channels; the bottom channel shows the calculated *R* values. (**b**) Histogram of *σ*^2^(*R*) for ~200 NP-liposomes containing DNA-NPs (top) or PEG-NPs (bottom) at pH=8. A two-sample *t*-test was applied to the data at log 10 scales and confirmed that the difference in distributions for DNA-NPs and PEG-NPs is highly significant (*P*<0.0001). DNA-NP, DNA-functionalized NP; NP, nanoparticle; PEG-NP, polyethylene glycol-functionalized NP.

As our simulations confirm that *R* is a useful spectral reporter of the interparticle separation, we set out to monitor the interactions of co-confined NPs by recording the *R* trajectories for individual NP-liposomes. Figure 4a contains 20 s long *I*(*λ*_1_) and *I*(*λ*_2_) trajectories of two DNA-NPs encapsulated in one liposome at pH=8 (top) and the computed *R*-trajectory (bottom). The trajectories vividly illustrate how the changes in *I*(*λ*_1_) and *I*(*λ*_2_) cause fluctuations in *R* and indicate a rich dynamics of the interparticle separation. For some NP-liposomes, the color shifts were sufficiently strong to be detectable by the eye and could be recorded with a conventional digital camera (Supplementary Movie S1). Not all recorded trajectories of liposome-confined DNA-NPs contain spectral fluctuations of this magnitude, but the DNA-NPs exhibit on average significantly more spectral fluctuations than control NPs without DNA. This phenomenon is demonstrated in Figure 4b, where we show a histogram of the variance *σ*^2^ of *R* for ~200 individual trajectories (20 s) of encapsulated DNA-NP dimers and PEG-NP controls. All data were recorded at pH=8. The NP-liposomes that contained DNA-NPs show a significantly broader *σ*^2^ distribution and a larger average *σ*^2^ value than the controls. We attribute the overall larger fluctuations in *R* for DNA-NPs at pH=8 to the presence of DNA-mediated transient binding contacts between the NPs that modulate the interparticle separation. Due to the distance dependence of plasmon coupling, a decrease in interparticle separation induced by a transient contact results in a spectral redshift. In our setup, spectral redshifts are detected by a higher *I*(*λ*_2_) (and lower *I*(*λ*_1_)) intensity and a reduced *R* value. Because repulsive interparticle interactions dominate for PEG-NPs, these transient contacts are missing for the control particles. Fluctuations in *R*, which result from transient binding interactions for DNA-NPs, and their absence in the case of PEG-NPs account for the observed differences in *σ*^2^(*R*) in Figure 4b.

**Figure 5:**
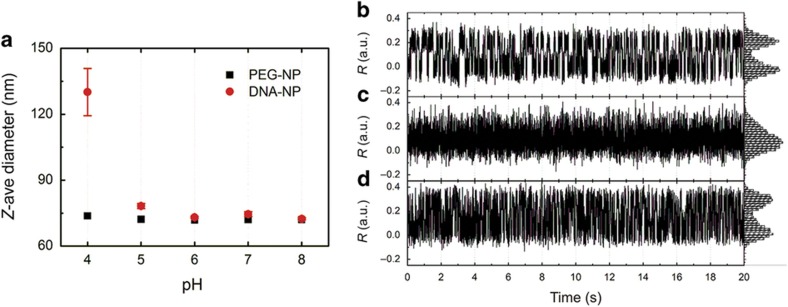
Modulation of C-rich DNA-NP interactions via pH. (**a**) *Z*-average diameter of DNA-NPs and PEG-NP controls in the pH range of 4–8 as determined by DLS. (**b**–**d**) *R* trajectories of an individual NP-liposome, which contained two DNA-NPs in an ambient medium of (**b**) pH=8, (**c**) pH=4, and (**d**) pH=8 again. Histograms of the *R* values are included on the *y* axis to illustrate the probability distribution of the *R* values. The *R* fluctuations exhibit a reversible pH-sensitive behavior. Both pH=8 measurements show a bimodal distribution with well separated high and low *R* value peaks, but the pH=4 histogram shows a single dominant peak at the lower end of the *R* value range. DLS, dynamic light scattering; DNA-NP, DNA-functionalized NP; PEG-NP, polyethylene glycol-functionalized NP.

The observed transient binding contacts between co-confined DNA-NPs in basic pH can arise from multiple weak base pairs between NPs carrying multiple identical DNA strands or short-lived bimolecular i-motif formation. The i-motif has a low stability at pH=8 (Refs. 43,45,46), but it is stabilized in acidic conditions when the pH approaches the p*K*_a_ (4.4) of cytosine^45,46,48^. Under these conditions, noncanonical C-C^+^ hydrogen bonds become more probable. In the next step, we investigated how a change in pH from basic (pH=8) to acidic (pH=4) affects the interparticle interactions of liposome-confined NPs with C-rich single-stranded DNAs. In bulk experiments, we observed that a decrease in pH is accompanied by an increase in hydrodynamic radius (measured by DLS) of DNA-NPs. This result is shown in Figure 5a, where the DNA-NPs have a hydrodynamic diameter of ~75 nm in the pH range of 5–8, which abruptly increases to 130 nm at pH 4. Interestingly, this increase was reversible, and increasing the pH value from 4 to 8 recovered the smaller hydrodynamic diameter. The bulk data confirm the presence of pH-induced interparticle attractions for the investigated DNA-NPs.

Consistent with these observations on the ensemble level, we detected a reversible association for DNA-NPs in some individual DNA-liposomes. The examples in Figures 5b–d show *R* trajectories for the same NP-liposome that contained two DNA-NPs in an ambient medium whose pH was gradually decreased from pH=8 to pH=4 in steps of pH=1, before it was increased to pH=8 again. We focus in Figures 5b–d on the *R* trajectories for pH=8, pH=4, and again pH=8 to illustrate the most distinct differences. For both pH=8 experiments, the spectral response shows *R* fluctuations between two distinct states, whereas for pH=4 the *R* value histogram is dominated by a single maximum at the lower end of the *R* value range. The observed repeated reversible *R* transitions between two distinct states at pH=8 indicate the binding and unbinding of the NPs that were co-confined to the interior of the liposome. In contrast, the stabilization of a low *R* value for pH=4 is congruous with a stabilization of the bound dimer state due to bimolecular C-C^+^ contact formation between the DNA-NPs. Interestingly, the *R* value for this bound state is slightly higher than that of the bound state for pH=8, which is indicative of systematic structural differences between the two bound states for the two pH values. Overall, the observed difference in spectral response between pH=8 and pH=4 implies that the C-rich DNAs on the NPs become more ‘sticky’ under acidic pH conditions and that the long-lived DNA-mediated NP contacts become energetically favored.

For pH=8, where the NPs experience a stochastic transition between the bound and non-bound states, we fitted the *R* trajectory to a two-state step function using a hidden Markov analysis (Supplementary Figure S1). From the fits to the dwell times (Supplementary Figure S2) of the bound and unbound states, we determined the association and disassociation rates *k*_off_ and *k*_on_ and the association constant *K*_A_=*k*_on_/*k*_off_. This analysis reveals *K*_A_ values of 1.04E7 and 3.10E6 for the trajectories in Figures 5b and d, respectively. The corresponding Gibbs free energies are as follows: Δ*G*°=−40.0 and −37.1 kJ mol^−1^. Based on these values, we estimate that the formation of ~5 bp contacts facilitated the intermittent binding between the two NPs at pH=8.

The differences in the measured dwell times for the two pH=8 measurements (Supplementary Figure S2) indicate that the acidification of the surface at low pH values has a small but measurable effect on the NP–NP interactions even after the pH is increased again. The small destabilization of the bound state can, for instance, result from a restructuring of surface-bound DNAs that have not yet equilibrated to the initial condition under our experimental conditions. The moderate but systematic shift in *R* between the two pH=8 measurements in Figures 5b and d is consistent with structural differences in the NP–NP contact in the two pH=8 trajectories.

We conclude that switching the pH between 4 and 8 strongly affects the interparticle potential of the co-confined NPs and that the precise structure of the bound state shows some variability for repeated changes of the ambient pH.

## Conclusion

We have used the distance-dependent spectral response of NPs that were co-confined in liposomes as transducer for interparticle interactions on deeply subdiffraction limit length scales. Unlike in conventional PR approaches, the NPs in the liposome-based system do not need to be covalently coupled as the spatial colocalization in a liposome retains the NPs in close contact without requiring a direct tether. The liposome encapsulation approach facilitates high local NP concentrations and significantly increases the probability of weak and transient binding contacts. Because the NP concentration is local, the approach enables the monitoring of NPs at the single-dimer level, which further aids the detection of short-lived binding contacts. We applied this technique to probe the interparticle interactions between NPs functionalized with C-rich DNAs and their dependence on pH. At pH=8, weak, transient interactions dominate, but we found experimental indications for the stabilization of C-C^+^-based i-motif-like contacts between the NPs at pH=4. The demonstrated ability to monitor transient interparticle interactions between individual pairs of non-bonded NPs—in principle without limitation in observation time—provides new opportunities for plasmon coupling in sensing and imaging. For example, it is easy to imagine how the pH dependence of the interparticle interactions between rationally designed DNA-NPs in optimized NP-liposomes can be exploited to monitor pH *in vitro* and *in vivo*. Furthermore, the ability to gather real-time spectral information that translates into interparticle distance information over extended periods of time is highly useful for detecting and characterizing transient particle binding events and their dependence on the ambient conditions. One application of relevance for cell analysis lies in the detection of specific metabolomes. When the binding of metabolomes changes the interparticle potential of adequately functionalized NPs, the NP-liposome approach is a sensitive strategy for their detection at low concentration. Importantly, the application space of NP-liposomes is not limited to sensing. Instead, the ability to switch reversibly the spectral response of individual NP-liposomes via chemical cues also paves the path to new nanophotonic switches.
