# Supplementary material for: Monitoring transient nanoparticle interactions with liposome-confined plasmonic transducers
Source: Microsyst Nanoeng. 2017 Apr 10;3:16086. doi: 10.1038/micronano.2016.86 (PMC5983364; doi:10.1038/micronano.2016.86)
Supplement: Supplementary Figures [file micronano201686-s1.pdf]

Supplementary file

Monitoring transient nanoparticle interactions with liposome-confined plasmonic transducers

Tianhong Chen, Xiao Wang, Mohammad Hossein Alizadeh and Björn M. Reinhard

Microsystems & Nanoengineering (2017) **3,** 16086; doi:10.1038/micronano.2016.86; Published online: 10 April 2017

DWELL TIME ANALYSIS

We performed dwell time analysis for the trajectories in Figures 5b and d in which two NPs co-confined within one liposome undergo stochastic transitions between a bound and unbound state. To that end, we fitted the *R* trajectories to a two-state step function using a hidden Markov approach. The analysis was performed with home-written Matlab codes using the Hidden Markov models as implemented in Matlab. Transition rates were determined from exponential fits to the dwell time distribution of the two states. The bimolecular association rate k_{on} was determined as

$$k_{on} = 1/\tau(\text{unbound}) * c_{NP}$$

where τ(unbound) is the characteristic decay time of the exponential fit of the unbound state and c_{NP} is the effective NP concentration of a dimer in a 200 nm diameter liposome.

The disassociation rate was determined as

$$k_{off} = 1/\tau(\text{bound})$$

where τ(bound) is the characteristic decay time of the exponential fit of the bound state.

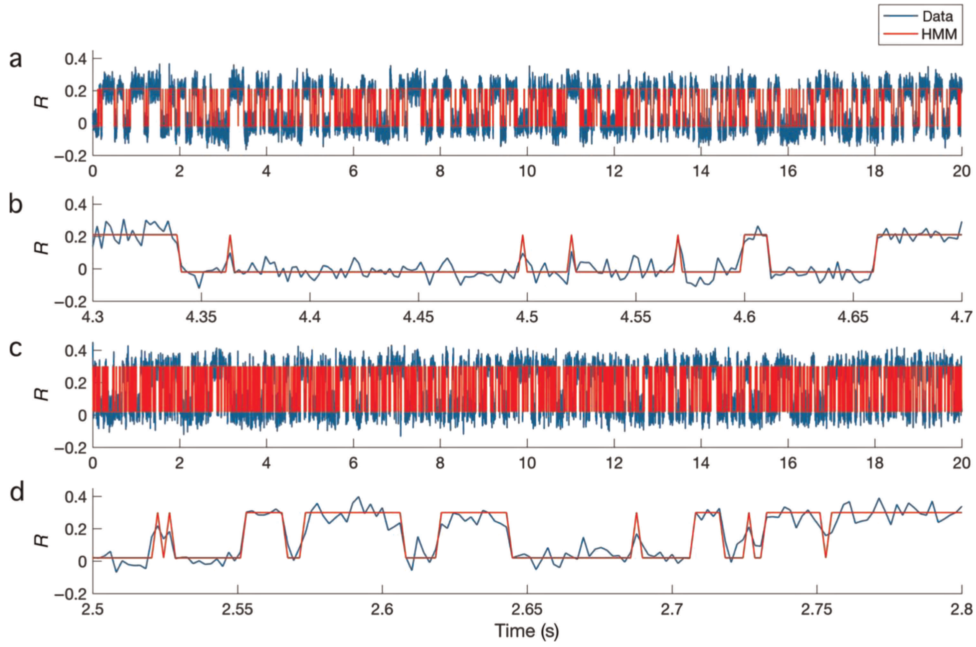

Figure S1 Hidden Markov Model (HMM) fitting of the pH 8 trajectories from Figures 5b and d of the main text. (**a**) and (**c**) show the entire trajectories and (**b**) and (**d**) show sections to highlight the two state fitting achieved through the HMM process. The high *R* state in the trajectories corresponds to the unbound state, the low *R* state to the bound state.

Department of Chemistry and The Photonics Center, Boston University, 590 Commonwealth Avenue, Boston, MA 02215, USA.
Correspondence: Björn M. Reinhard (bmr@bu.edu)

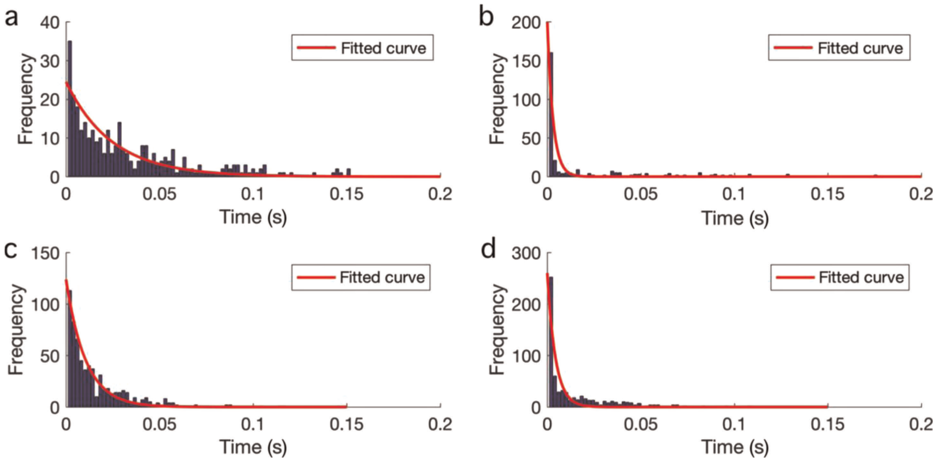

Figure S2 Dwell time distributions and exponential fits for bound (low *R*) and unbound (high *R*) states for the two pH 8 trajectories shown in Figure S1. (**a**) and (**c**) show the dwell time distributions for the bound states in Figures S1a and c, respectively. (**b**) and (**d**) show the distributions for the unbound state in Figures S1a and c. The fitted τ values are: (**a**) 0.0247 s; (**b**) 0.0031 s; (**c**) 0.0105 s; (**d**) 0.0044 s.
